# Epizootic Yersinia enterocolitica in captive African green monkeys (*Chlorocebus aethiops sabaeus*)

**DOI:** 10.3389/fvets.2022.922961

**Published:** 2022-11-23

**Authors:** Gayathriy Balamayooran, Hannah M. Atkins, Rachel N. Andrews, Kristofer T. Michalson, A. Robert Hutchison, Andre C. LeGrande, Quentin N. Wilson, Melaney K. Gee, S. Tyler Aycock, Matthew J. Jorgensen, Richard W. Young, Nancy D. Kock, David L. Caudell

**Affiliations:** Section on Comparative Medicine, Department of Pathology, Wake Forest School of Medicine, Winston-Salem, NC, United States

**Keywords:** non-human primate, enterocolitis, lymphadenitis, typhlitis, yersiniosis, immunophenotype, vervet/African green monkeys

## Abstract

*Yersinia enterocolitica* is a Gram-negative bacterium that typical results in enterocolitis in humans and poses significant worldwide risks to public health. An outbreak of yersiniosis in the Vervet/African green monkey colony at the WFSM during the winter of 2015–2016 accounted for widespread systemic infection with high morbidity and mortality. Most of the cases had extensive necrosis with suppuration and large colonies of bacilli in the large bowel and associated lymph nodes; however, the small intestine, stomach, and other organs were also regularly affected. Positive cultures of *Yersinia enterocolitica* were recovered from affected tissues in 20 of the 23 cases. Carrier animals in the colony were suspected as the source of the infection because many clinically normal animals were culture-positive during and after the outbreak. In this study, we describe the gross and histology findings and immune cell profiles in different organs of affected animals. We found increased numbers of myeloid-derived phagocytes and CD11C-positive antigen-presenting cells and fewer adaptive T and B lymphocytes, suggesting an immunocompromised state in these animals. The pathogen-mediated microenvironment may have contributed to the immunosuppression and rapid spread of the infection in the vervets. Further studies in vervets could provide a better understanding of *Yersinia*-mediated pathogenesis and immunosuppression, which could be fundamental to understanding chronic and systemic inflammatory diseases in humans.

## Introduction

Yersiniosis caused by *Yersinia enterocolitica* is an important food-borne zoonosis with substantial importance to the public health. *Y.enterocolitica* is a Gram-negative, facultative anaerobic bacillus with a worldwide distribution (1, 2). The disease is often limited to the alimentary system, but can also result in systemic infection. Infection occurs after ingesting contaminated milk, raw or undercooked meat, fresh vegetables, or water. Symptoms of yersiniosis range from self-limiting suppurative enterocolitis to severe mesenteric lymphadenitis or sepsis. Sepsis is a particular risk in older or immunocompromised populations (3).

*Y.enterocolitica* demonstrates high antigenic variability. The most pathogenic strains associated with disease in humans are 1B/O:8, 2/O:5, 2/O:9, 3/O:3, and 4/O:3 with 1B/O:8 frequently isolated in North America. In non-human primates, O8, O3, O5/27 and O9 have been reported to cause disease (4).

In non-human primates, disease outbreaks due to *Y. enterocolitica* infection have been reported in African green monkeys, squirrel monkeys, gibbons, and patas monkeys (2, 4–6). *Y. enterocolitica* has been isolated from many birds and mammals, yet pigs are considered the primary reservoir for pathogenic human strains (7–10). The primary mode of transmission is fecal-oral among captive animal populations, and contamination of food and water sources has been well-documented (11, 12). Wild rodents are latent reservoirs for *Y. enterocolitica* and potential disease carriers (13, 14).

Antibiotics remain the mainstay of treatment. However, harmful side effects of antibiotics, increasing antibiotic resistant strains and increasing immunocompromised population warrants better treatment modalities and mucosal vaccine. Studying a natural infection in non-human primates would provide better understanding of the disease and aid in development of better therapeutics.

At Wake Forest School of Medicine, two out breaks occurred in the African green monkeys (vervets/*Chlorocebus aethiops sabaeus*) breeding colony. During the 2015 outbreak, all had systemic disease, in contrast to the 2011 outbreak, in which only the intestine was affected. Here, we describe the gross, microscopic findings enriched with alteration in immune cell phenotype in vervets during the 2015 yersiniosis outbreak.

## Materials and methods

### Animal care and husbandry

Over 300 African green monkeys were housed in two adjacent buildings, with eight breeding pens per building. The Wake Forest School of Medicine (WFSM) is accredited by the Association for the Assessment and Accreditation of Laboratory Animal Care (AAALAC) and is compliant with the Animal Welfare Act. The WFSM Animal Care and Use Committee approved all research protocols involving animals and performed following the Guide for Care and Use of Laboratory Animals (15). During the outbreak, the colony was fed standard laboratory monkey chow (LabDiet 5038, Purina, St. Louis, MO) daily and given enrichment 5 days a week from commercial sources and an on-site garden located several yards from the colony. Water was provided by the City of Winston-Salem municipal water service. The WFSM Animal Resources Program (ARP) provided care for the animals, and there were no changes in husbandry or food enrichment before or during the outbreak. In September 2015, annual physical examinations were performed, at which time cases of yersiniosis were not detected.

#### Case history

In 2011, a small, self-limiting outbreak of enteric yersiniosis occurred in an indoor-outdoor breeding colony of African green monkeys at the Wake Forest School of Medicine, during which four animals died (16). The macroscopic and microscopic lesions in those cases almost exclusively involved the intestines. This research colony was transferred from California in 2008, and rare cases of yersiniosis occurred before relocation.

Four years after the 2011 outbreak, a second series of enteric yersiniosis occurred from November 2015 through March 2016. Twenty-three animals were found dead, died during treatment, or required euthanasia because of advanced disease. Many animals were asymptomatic until the day of death or euthanasia, at which time many had firm palpable abdominal masses and swollen rectums. Diarrhea was infrequent, with constipation being more common in advanced cases. Most cases occurred in animals under 3 years of age, but adults were also regularly affected Yersiniosis was relatively widespread within the colony as fecal bacterial cultures collected during the outbreak revealed an infection prevalence of 30% (92/304 animals) in the remaining live vervets, despite aggressive preventative measures taken by the clinical care staff.

Treatment strategies varied, but initial therapy included removing clinically ill individual animals to single-housing and treating with intramuscular administration of enrofloxacin and ceftiofur for 14 days, daily subcutaneous fluids (Lactated Ringer's solution or 0.9% saline) for 3 days, and meloxicam for 3 days, given either subcutaneous or orally.

As the number of cases grew, a more feasible herd-based approach was implemented. This strategy involved moving all the animals from each breeding group out of the large enclosures into rack-style caging in a different building for isolation and to provide easier access for observation and treatment. There animals were treated with enrofloxacin for 10 days and later, gentamicin proved to be a reliable alternative and required a shorter effective therapeutic course of 5 days. Increased fluid intake was provided by offering orange slices daily and administration of subcutaneous fluids to those detected to be dehydrated based on clinical observation.

All animals were tested by PCR screening of feces for *Yersinia enterocolitica* (VRL Labs, San Antonio) A negative result for all members of each group was required prior to returning to the home enclosure.

### Necropsy

All animals were either found dead or humanely euthanized with sodium pentobarbital. Each underwent a complete necropsy examination with collection of heart, lung, liver, kidney, spleen, skin, reproductive organs, lymph nodes, brain, eyes, endocrine glands, alimentary canal, and bone marrow into 10% neutral buffered formalin. After fixation for at least 24 h, the tissues were trimmed, embedded in paraffin, sectioned at 4 μm, stained with hematoxylin and eosin (HE), and evaluated by light microscopy by one of three faculty veterinary pathologists. In some cases, affected tissues were also examined with Gram stains.

#### Bacterial culture

Bacterial cultures were obtained from grossly affected organs at necropsy in 22/23 cases and submitted to IDEXX Laboratories, Inc (North Grafton, MA) for aerobic and anaerobic culture. Water sources and environmental surfaces before and after cleaning from affected pens were sampled and submitted to QUIP Laboratories (Wilmington, DE) for bacterial culture using TSA, 5% sheep blood agar, R2A, PDA, and SMA media for water samples, and Difco HYcheck slides (Becton Dickinson and Company, Franklin Lakes, NJ) for environmental surface samples. Any growth after 24 h was submitted to MIDI Labs (Newark, DE) for *Y. enterocolitica* sub-culture and identification using Matrix Assisted Laser Desorption Ionization Time-Of-Flight (MALDI-TOF) mass spectrometry.

#### Immunohistochemistry

To evaluate the immune system's reaction to *Y. enterocolitica* infection, sections of lymph node, placenta, lung, and colon from infected vervets were stained for T cells (anti-CD3, Dako, A0452, clone F7.2.38, 1:300 dilution), B cells (anti-CD20cy, Dako, M0755, clone L26, 1:1500), antigen-presenting cells (anti-CD11c, Leica, NCL-L-CD11c-56, clone 5D11, 1:200), neutrophils (anti-myeloperoxidase, MPO, Leica, NCL-L-MYELO, clone 59A5, 1:50), and macrophages (Sigma, 279M-16, clone HAM56, 1:50). Clinically healthy control tissues from age-matched animals were also stained for CD3, CD20, CD11c, MPO, and HAM56 and compared to infected tissues. All immunohistochemistry was performed using an automated staining system (Leica Biosystems Inc., Buffalo Grove, IL). The chromogen was Fast Red (Leica Biosystems Inc.), and tissues were counterstained with Mayer's hematoxylin. Positive and negative immunohistochemical controls were run in conjunction with test samples. Finally, slides were imaged using a BX61VS Olympus microscope (Olympus Corporation, Waltham, MA). Two veterinary pathologists evaluated immunohistochemical staining independently and assigned a value from negative (-) to positive staining (+ to +++).

## Results

### Gross pathology

**Figure 1:**
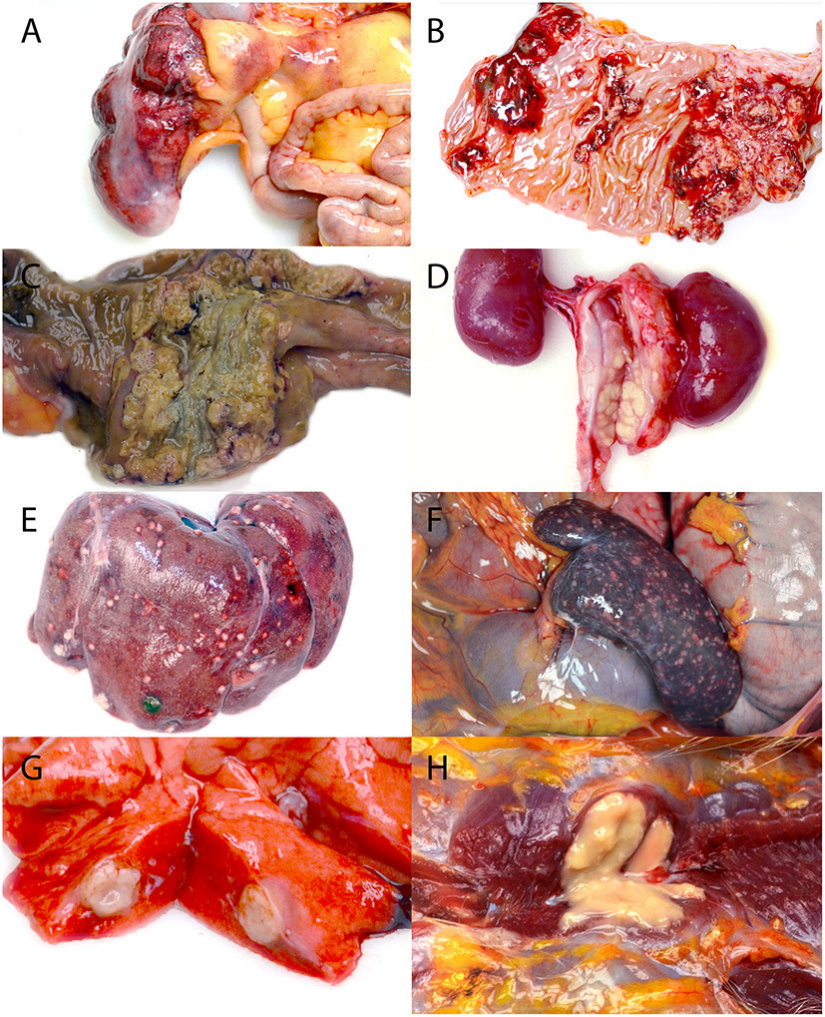
Gross lesions associated with the systemic yersiniosis epizootic, vervets, multiple organs. **(A)** Typhlitis, cecum, case 17. Bright red hemorrhages diffusely cover the serosal surface of the colon, and the wall is markedly thickened. **(B)** Gastritis, stomach, case 15. The mucosa is ulcerated and hemorrhagic in several coalescing regions. **(C)** Colitis, colon, case 17. A yellow to green-gray fibrinonecrotic pseudomembrane covers the ulcerated colonic mucosa. **(D)** Lymphadenitis, renal lymph node, case 10. Markedly enlarged renal lymph nodes are replaced by yellow caseous material. **(E)** Hepatitis, liver, case 18. Off-white nodules up to 3 mm in diameter are present on the surface of the liver. **(F)** Splenitis, spleen, case 13. The spleen is enlarged, and myriad, tan to red, 0.5–1 mm diameter foci are scattered across the surface. **(G)** Pneumonia, lung, case 8. A 1 cm diameter abscess effaces part of a middle lung lobe. **(H)** Myositis, right pectoralis muscle, case 16. A 1.5 cm diameter abscess expands and replaces the muscle.

Twenty-two of the animals presented acutely moribund or were found dead on the day of necropsy, all with necrotizing, often transmural, inflammation of the cecum, colon, or rectum with extension into the small intestine and stomach in some cases (Figures 1A–C). The affected areas were friable and discolored dark red to green. In the most severe cases, the cecal and colonic walls were expanded by inflammatory exudate, necrotic debris, fibrin, and hemorrhage, which occluded as much as 80% of the lumina. Mucosal ulceration ranged from pinpoint to regionally extensive. Larger ulcers were sometimes overlain by adherent fibrinonecrotic pseudomembranes (Figure 1C). The cause of what was suspected to be abdominal mass lesions in many animals was adhesive peritonitis of varying chronicity, which sometimes required blunt dissection of the omentum and adjacent viscera from the cecum and colon. Enlargement and variable effacement of the mesenteric lymph nodes by suppurative to caseous material occurred in 15 cases (65%), with similar changes consistently present in the colonic and rectal lymph nodes and less often in the pancreatic, splenic, and perirenal lymph nodes (Figure 1D).

The liver (12/23, 52%), spleen (10/23, 43%), and lung (8/23, 35%) were the most frequently affected organs aside from the gastrointestinal tract, all of which had firm, off-white to yellow, 1 mm−2 cm diameter abscesses (Figures 1E–G). Renal abscessation was visible in one case. Other cases included suppurative inflammation extending from the adductor muscles into the bone marrow of the pubic symphysis, suppurative pectoral myositis (Figure 1H), suppurative osteomyelitis of the femur, and suppurative placentitis without fetal lesions. The index case was a 2-month-old male vervet with severe, necrotizing bronchointerstitial pneumonia and ulcerative tracheitis without gastrointestinal involvement. Gross findings are summarized in Table 1.

**Table 1:** Summary of gross lesions by organ system in African green monkeys infected with *Yersinia enterocolitica*^*a*^.

**Case**	**Age**	**Sex**	**Pulmonary**	**Urogenital**	**Hemolymphatic**	**Hepatobiliary**	**GI**	**Musculoskeletal**
1	0.2	M	+++^*^	-	-	+	-	-
2	0.5	M	-	-	+++	++	+++^*^	-
3	0.6	M	+	-	+++^*^	-	+++	-
4	0.6	M	-	-	+++^*^	++	+++	-
5	0.6	M	-	-	++^*^	-	+++	-
6	0.6	M	+++	-	+^*^	+	+++	-
7	0.6	M	-	-	++^*^	-	++	-
8	0.7	F	++	-	+++^*^	-	+++	-
9	1.5	F	-	-	+++^*^	-	+++	-
10	2.2	F	-	-	++^*^	++	+++	-
11	2.3	M	-	-	+++^*^	+	+++	-
12	2.5	M	++	-	+	++	+++	-
13	3.6	F	-	-	+++^*^	-	+++	-
14	3.8	F	-	-	+++	-	+++	-
15	6.6	M	-	-	-*	-	+++	-
16	6.6	F	++^*^	-	++	+++	+++^*^	++^*^
17	7.3	F	-	-	+++^*^	-	+++	-
18	7.5	F	-	-	++^*^	++	+++	-
19	8.4	F	++^*^	+	++	+	+++	-
20	8.6	F	++	+++	++	++	+++	-
21	14.4	F	-	-	-	-	+++^*^	-
22	15.5	F	-	-	-	-	+++	-
23	22.3	F	-	-	++	++^*^	++	-

a Cases are ordered by age in years. Lesions were scored for severity (- no lesions; + mild; ++ moderate; +++ severe). F, Female; M, Male; GI, Gastrointestinal system.

* Denotes positive Y enterocolitica culture.

### Histopathology

**Figure 2:**
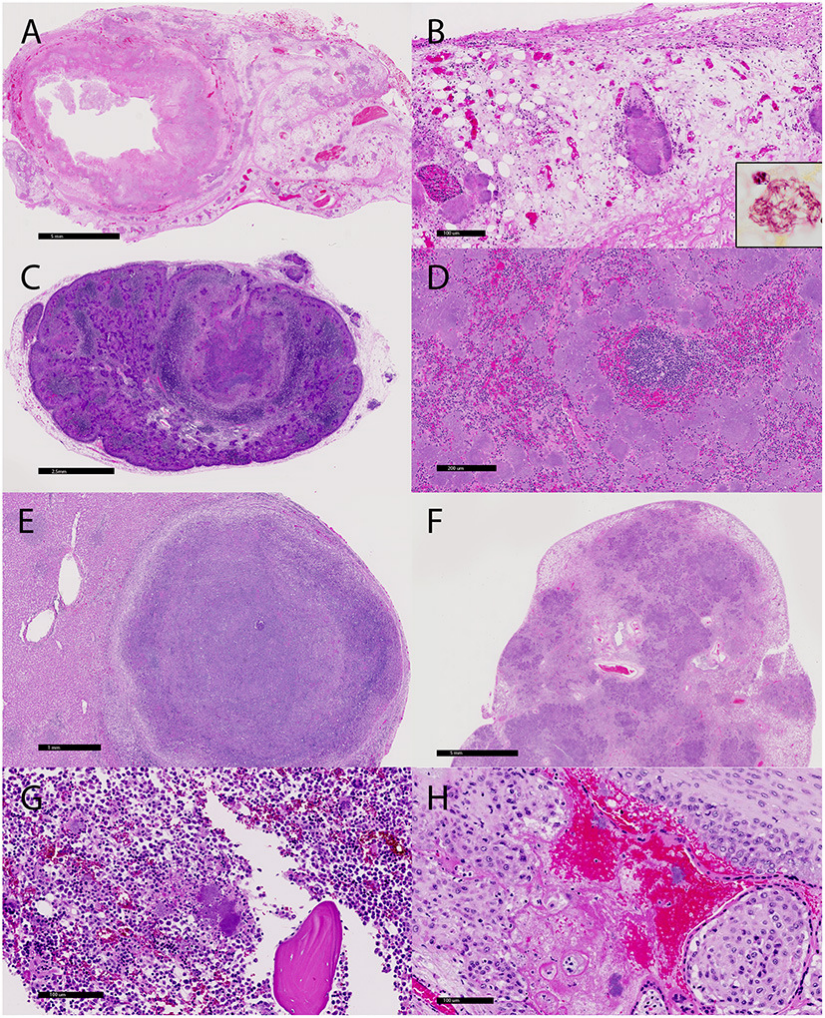
Microscopic lesions associated with the yersiniosis epizootic, vervets, multiple organs. **(A)** Colitis, colon, case 3. The colonic wall is transmurally necrotic and expanded by inflammatory cells. Large colonies of bacilli extend into lymphatic vessels and the adjacent mesentery. HE. **(B)** Colon, case 3. Large colonies of bacilli are present in the mesentery. Inset: A colony of Gram-negative bacilli within the necrotic debris. Gram histochemical stain. **(C)** Lymphadenitis, mesenteric lymph node, case 18. Large bacterial colonies and necrotic debris efface the normal lymph node architecture. HE. **(D)** Splenitis, spleen, case 5. Colonies of 1x5 μm bacilli largely obliterate the red pulp. **(E)** Hepatitis, liver, case 19. A well-demarcated abscess effaces the parenchyma. HE. **(F)** Pneumonia, lung, case 1. The lung architecture is extensively obscured by adjoining regions of bacteria, inflammation, and necrosis. HE. **(G)** Osteomyelitis, femur, case 18. Large colonies of bacteria, degenerate neutrophils, and cellular debris surround a fragment of necrotic bone in the medullary cavity. HE. **(H)** Placentitis, placenta, case 20. Bacteria and neutrophils infiltrate the decidua and maternal sinusoids. HE.

Fibrinosuppurative inflammation affected the large bowel in 22 of the 23 cases (96%), many with marked expansion of the lamina propria and submucosa, ulceration, submucosal abscessation, and some with transmural effacement (Figure 2A). Large colonies of Gram-negative 1 x 5 μm bacilli were typically observed within the necrotic foci and throughout depleted lymphoid follicles. Many lymphatic vessel lumina, particularly along the serosal edges of the affected regions and throughout the adjacent mesentery, were filled with bacteria (Figure 2B and inset). Small intestinal and gastric lesions were present in four and two cases, respectively, but neutrophilic infiltration of the serosal surfaces, mesentery, and regional lymph nodes was common. Regional to complete effacement of mesenteric lymph nodes by suppurative inflammation with abundant intralesional bacilli was striking (Figure 2C).

Suppurative lesions outside of the gastrointestinal tract with large colonies of bacilli occurred in 16 cases (70%), with the spleen (11/23, 48%), liver (14/23, 61%), and lung (10/23, 43%) most commonly affected. Affected spleens had a marked reduction in the white pulp and up to 80% effacement by necrosis and large bacterial colonies (Figure 2D). Hepatic and pulmonary lesions typically ranged from microscopic sites of suppurative inflammation to large, encapsulated abscesses (Figures 2E,F). The lung of the index case with bronchointerstitial pneumonia was regionally effaced by necrotizing inflammation teeming with bacteria (Figure 2F). Other lesions in individual cases included suppurative skeletal myositis, femoral osteomyelitis (Figure 2G), and placentitis (Figure 2H). Several freshly necropsied animals had widespread bacterial emboli consistent with *Y. enterocolitica* in capillaries. Table 2 summarizes the microscopic findings.

**Table 2:** Summary of histologic lesions by organ system in African green monkeys infected with *Yersinia enterocolitica*^*a*^.

**Case**	**Age**	**Sex**	**Pulmonary**	**Urogenital**	**Hemolymphatic**	**Hepatobiliary**	**GI**	**Musculoskeletal**
1	0.2	M	+++	++	++	+++	-	-
2	0.5	M	+	+	+++	+	+++	-
3	0.6	M	-	-	+++	-	+++	-
4	0.6	M	-	+	+++	++	+++	-
5	0.6	M	-	+	+++	++	+++	-
6	0.6	M	+++	-	+++	+++	+++	-
7	0.6	M	-	-	++	-	+++	-
8	0.7	F	++	-	+++	-	+++	-
9	1.5	F	-	-	++	-	+++	-
10	2.2	F	-	-	++	+++	+++	-
11	2.3	M	-	-	+++	+++	++	-
12	2.5	M	++	-	++	++	+++	-
13	3.6	F	-	-	+++	-	+++	-
14	3.8	F	-	-	++	-	+++	-
15	6.6	M	-	-	-	-	+++	-
16	6.6	F	++	-	+++	+++	+++	+++
17	7.3	F	-	++	+++	-	+++	-
18	7.5	F	+	-	+++	++	+++	+
19	8.4	F	+++	++	+++	+++	+++	-
20	8.6	F	++	+	+++	++	+++	-
21	14.4	F	+	++	+	+	+++	-
22	15.5	F	-	-	+	-	+++	-
23	22.3	F	-	-	+++	+++	++	-

a Cases are ordered by age in years. Lesions were scored for severity (- no lesions; + mild; ++ moderate; +++ severe). F, Female; M, Male; GI, Gastrointestinal system.

### Microbiology

Twenty of the 23 cases cultured positive for *Y. enterocolitica*. Positive results were obtained from several different organ systems (Table 1), including the pulmonary (*n* = 3), hemolymphatic (*n* = 13), hepatobiliary (*n* = 1), gastrointestinal (*n* = 3), and musculoskeletal (*n* = 1) systems. Positive cultures were not obtained from water and environmental surfaces in the affected pens.

### Immunohistochemistry

**Figure 3:**
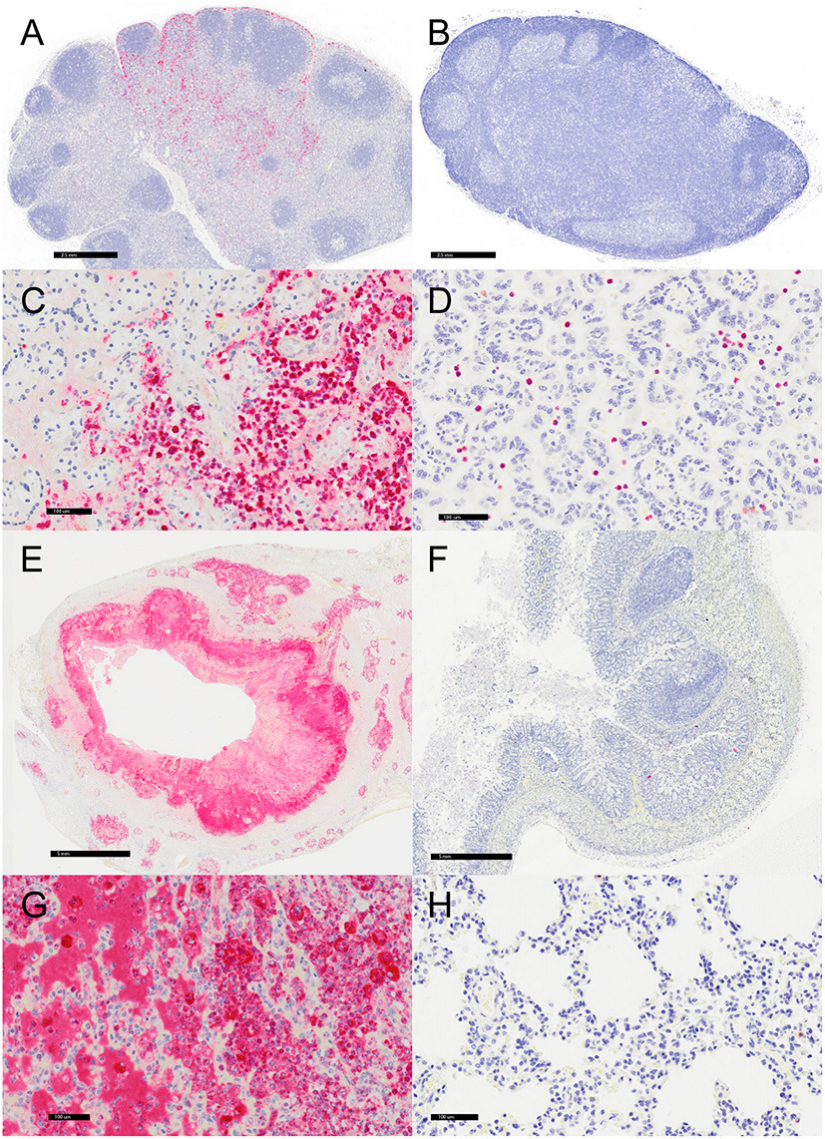
Immunohistochemistry for myeloperoxidase (MPO)-positive neutrophils, vervets, multiple organs. **(A)** *Y. enterocolitica-*positive vervet, mesenteric lymph node, case 6. Neutrophils are present within the medullary sinus of a mesenteric lymph node adjacent to the affected colon. **(B)** Control vervet, mesenteric lymph node. Neutrophils are rarely observed within the control lymph node. **(C)** *Y. enterocolitica-*positive vervet, placenta, case 20. MPO-positive neutrophils are abundant within areas of placental necrosis. **(D)** Control vervet, placenta. Few neutrophils are present within the placenta, and often associated with physiologically normal areas of coagulative necrosis and mineral. **(E)** *Y. enterocolitica-*positive vervet, colon, case 3. MPO-positive neutrophils are prominent within mucosal lesions. Neutrophils are also present within veins and lymphatic vessels of the muscularis mucosa. **(F)** Control vervet, colon. Neutrophils within the control colon are rare and mainly within the lamina propria. **(G)** *Y. enterocolitica-*positive vervet, lung, case 1. Extravasated neutrophils are abundant within lesions of the lung. **(H)** Control vervet, lung. MPO-positive neutrophils are present only within blood vessels of the control lung.

**Figure 4:**
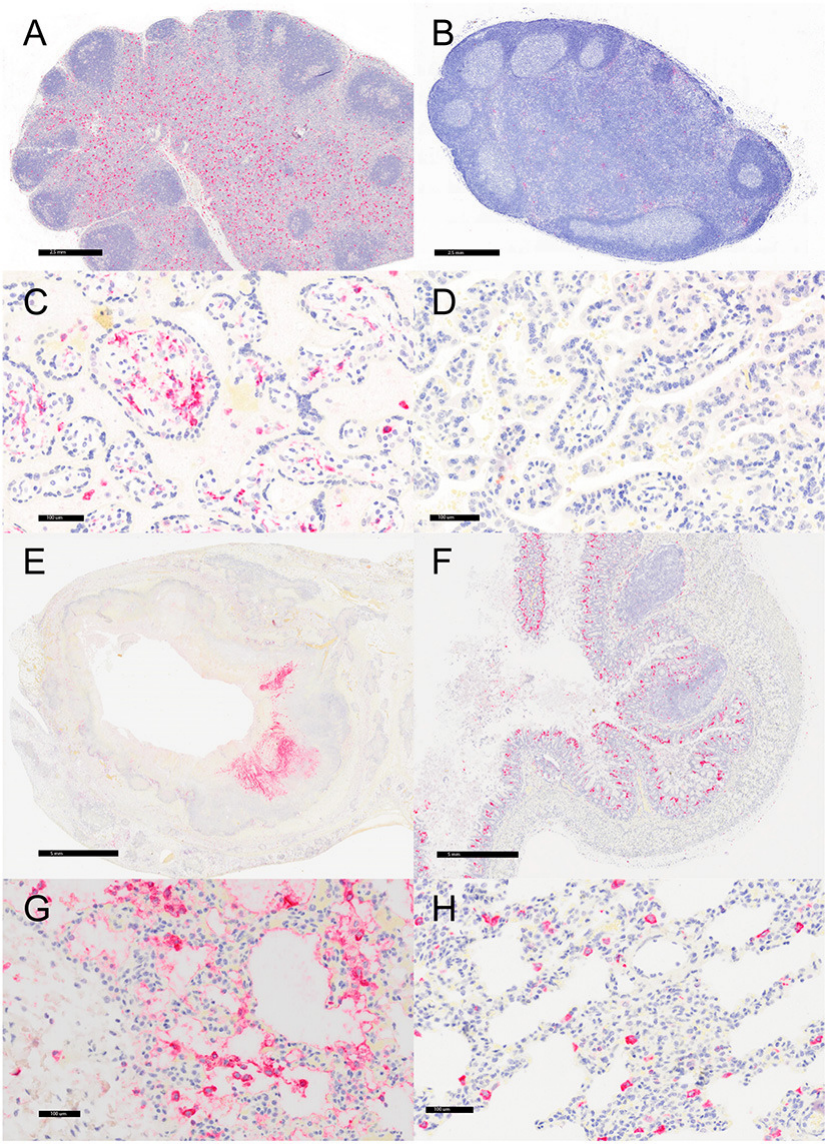
Immunohistochemistry for HAM56, vervets, multiple organs. **(A)** *Y. enterocolitica-*positive vervet, mesenteric lymph node, case 6. About 40% of the cells within the medullary sinus are HAM56-positive macrophages. This lymph node was adjacent to a colonic lesion. **(B)** Control vervet, mesenteric lymph node. A control lymph node contains few HAM56-positive macrophages (<10%). **(C)** *Y. enterocolitica-*positive vervet, placenta, case 20. Abundant HAM56-positive macrophages are present in fetal villi and also within maternal blood. **(D)** Control vervet, placenta. HAM56-positive macrophages are absent in the control placenta. **(E)** *Y. enterocolitica-*positive vervet, colon, case 3. HAM56-positive macrophages are present adjacent to necrosis. **(F)** Control vervet, colon. Macrophages are distributed throughout the colonic mucosa. **(G)** *Y. enterocolitica-*positive vervet, lung, case 1. Macrophages are prominent in alveoli at the periphery of necrotic lesions. **(H)** Control vervet, lung. HAM56-positive macrophages are scattered within alveolar walls.

**Figure 5:**
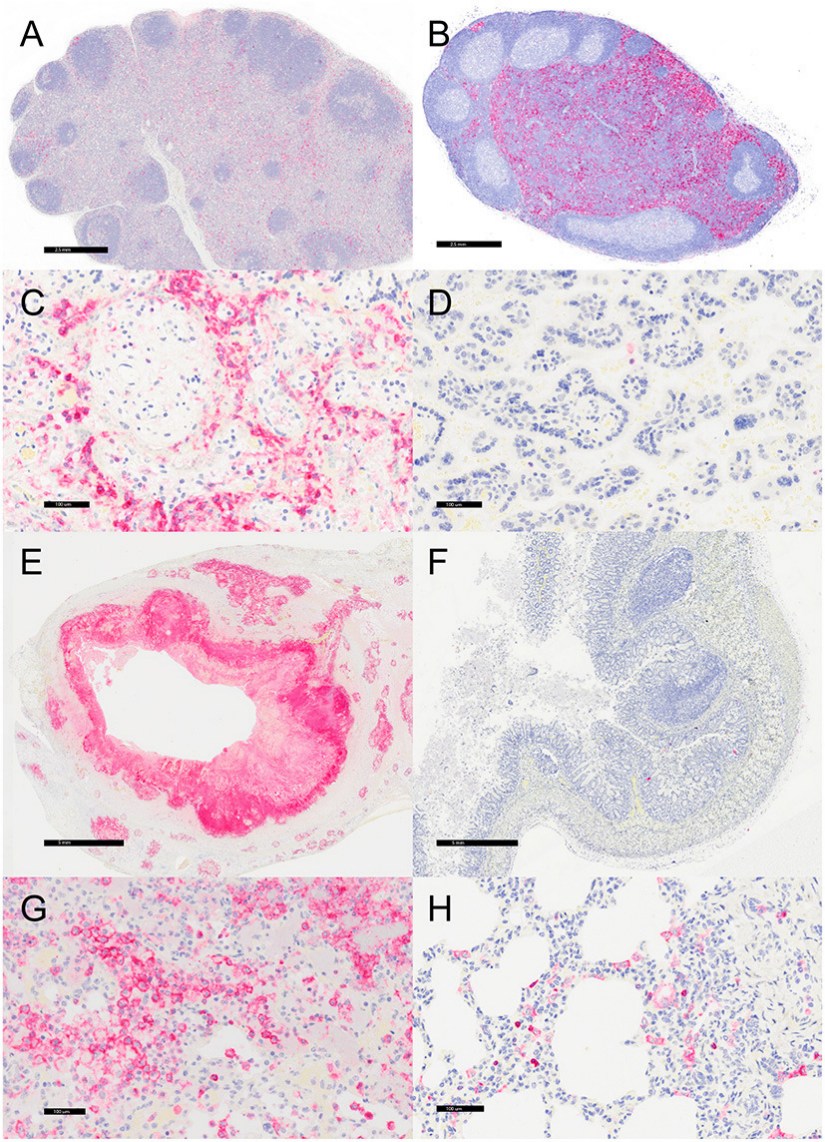
Immunohistochemistry for CD11c, vervets, multiple organs. **(A)** *Y. enterocolitica-*positive vervet, mesenteric lymph node, case 6. Approximately 20% of cells within the lymph node medullary sinus stain positively for CD11c. **(B)** Control vervet, mesenteric lymph node. CD11c-positive antigen-presenting cells (APCs) make up about 80% of the cells within the cortex and medullary sinuses of the lymph node. **(C)** *Y. enterocolitica-*positive vervet, placenta, case 20. CD11c-positive APCs are centered on areas of necrosis and are also increased in maternal blood. **(D)** Control vervet, placenta. CD11c-positive cells are rare within the control placenta. **(E)** *Y. enterocolitica-*positive vervet, colon, case 3. CD11c-positive cells are prevalent within lesions. **(F)** Control vervet, colon. Two CD11c-positive APCs are present within the colonic mucosa. **(G)** *Y. enterocolitica-*positive vervet, lung, case 1. Abundant CD11c-positive cells are found within necrotic lesions. **(H)** Control vervet, lung. CD11c-positive cells are numerous but confined to the interstitial space of the control lung.

**Figure 6:**
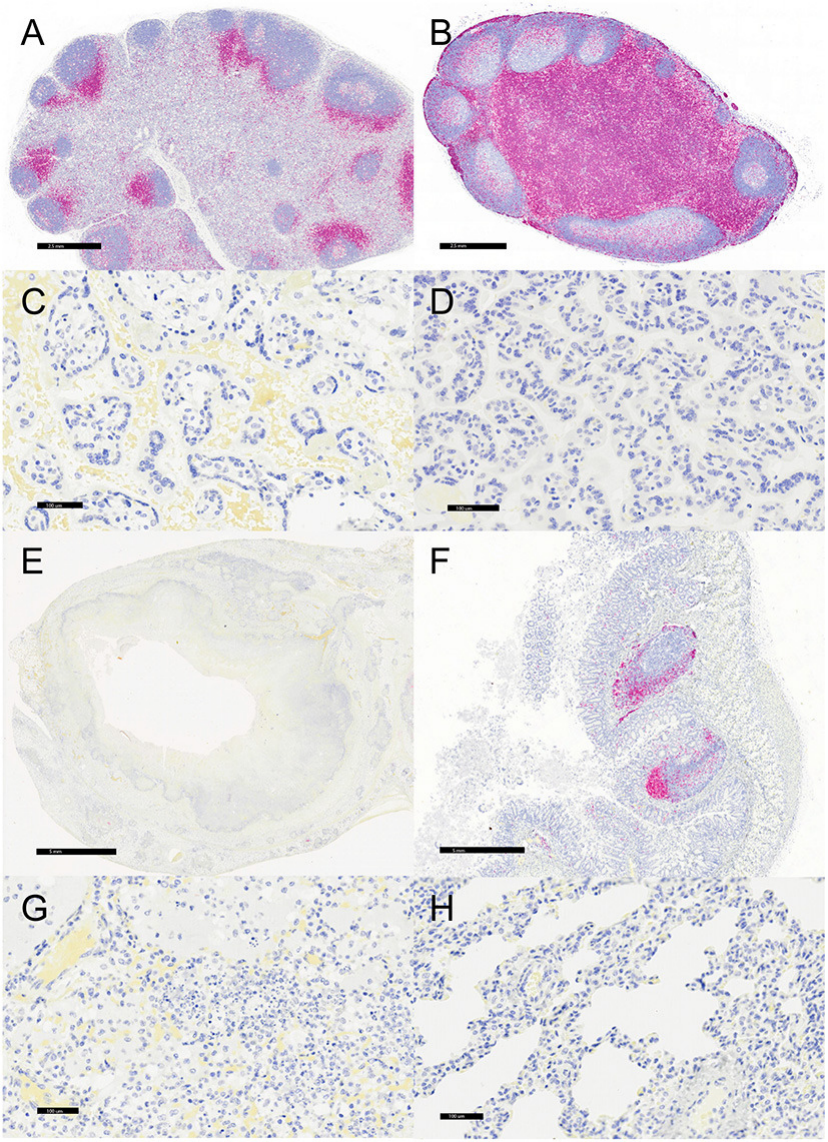
Immunohistochemistry for CD3, vervets, multiple organs. **(A)** *Y. enterocolitica-*positive vervet, mesenteric lymph node, case 6. Few CD3-positive cells are present within the lymph node medulla, with the remaining cells confined to perifollicular regions of the cortex. **(B)** Control vervet, mesenteric lymph node. Greater than 90% of cells within the lymph node are CD3-positive T cells. **(C)** *Y. enterocolitica-*positive vervet, placenta, case 20. CD3-positive T cells are absent in the placenta from a *Y. enterocolitica-*positive vervet. **(D)** Control vervet, placenta. CD3-positive T cells are absent from a gestational date-matched control in the placenta. **(E)** *Y. enterocolitica-*positive vervet, colon, case 3. CD3-positive T cells are absent from colonic sections as lesions effaced gut-associated lymphoid tissue. **(F)** Control vervet, colon. CD3-positive lymphocytes are present within gut-associated lymphoid tissue and colonic mucosa. **(G)** *Y. enterocolitica-*positive vervet, lung, case 1. CD3-positive T cells are absent in lung sections. **(H)** Control vervet, lung. T cells are also absent in an age-matched area of control lung.

**Figure 7:**
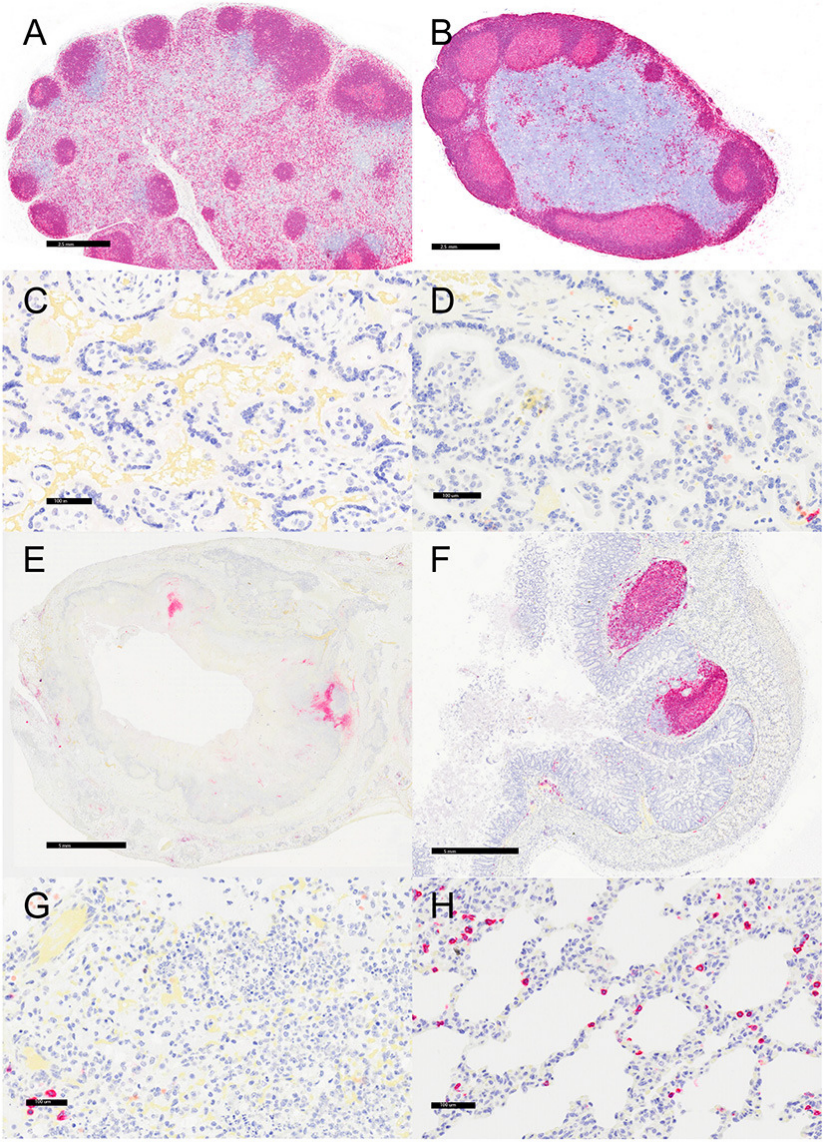
Immunohistochemistry for CD20, vervets, multiple organs. **(A)** *Y. enterocolitica-*positive vervet, mesenteric lymph node, case 6. CD20-positive B cells are present in follicles and the medullary sinus. **(B)** Control vervet, mesenteric lymph node. CD20-positive cells are most prevalent in the cortical follicles. **(C,D)** CD20-positive cells are rare in the placenta from a *Y. enterocolitica-*positive vervet (C, case 20) and in the control vervet **(D)**. **(E)** *Y. enterocolitica-*positive vervet, colon, case 3. CD20-positive B cells are present in the colonic mucosa in areas of presumed gut-associated lymphoid tissue that are mostly effaced by necrosis and suppurative inflammation. **(F)** Control vervet, colon. CD20-positive B lymphocytes are abundant within gut-associated lymphoid tissue and rare in colonic mucosa. **(G)** *Y. enterocolitica-*positive vervet, lung, case 20. Few CD20-positive B cells lie adjacent to areas of necrosis. **(H)** Control vervet, lung. B cells are more prevalent and scattered throughout the alveolar walls of the control lung.

Representative sections of the colon, lymph node, lung, and placenta in animals infected with *Y. enterocolitica* were immunophenotyped and compared to age-matched control animals (Table 3). The inflammation was primarily neutrophilic, confirmed by MPO immunostaining (Figure 3). Neutrophils were regionally distributed within the lymph node subcapsular, paracortical, and medullary sinuses (Figure 3A; neg. control Figure 3B), placental intervillous spaces (Figure 3C; neg. control Figure 3D), and throughout the inflammatory infiltrate in both lung (Figure 3E; neg. control Figure 3F) and colon (Figure 3G; neg. control Figure 3H). HAM56 distribution was similar, although more sparsely scattered. Medullary sinus histiocytosis was evidenced in lymph nodes (HAM56^+^ cells). HAM56 positive macrophages were associated with areas of necrosis within the colon (Figure 4). *Y. enterocolitica* infection was also associated with increased antigen-presenting cells (CD11c positive) within the lymph node, placenta, colon, and lung (Figure 5). CD3-positive T-lymphocytes were markedly decreased throughout the lymph node, but there was no difference in T lymphocyte staining in the placenta, colon, or lung (Figure 6). CD3 immunoreactivity was absent in the Yersinia positive colon (Figure 6E), and gut-associated lymphoid tissue was absent. Fewer CD20-positive B cells were present in mesenteric lymph node follicles (Figure 7A) but were increased in the lymph node medullary sinus compared to the healthy control (Figure 7B). CD20 immunoreactivity was decreased in *Y. enterocolitica*-positive sections of the placenta, colon, and lung primarily due to loss of mucosal-associated lymphoid tissue (Figures 7C–H). The mesenteric lymph node from a *Yersinia-*positive animal represented in Figures 3A, 4A, 5A, 6A, 7A was obtained from an area adjacent to a colonic lesion. It was negative for bacteria on Gram stain (Gram stain not shown).

**Table 3:** Immunohistochemistry of selected *Yersinia enterocolitica* positive and age-matched negative control tissues^a^.

**Case**	**Necropsy #**	**Y. +/-**	**Tissue**	**CD3**	**CD20**	**MPO**	**HAM56**	**CD11c**
20	16030	+	Placenta	-	-	+++	++	++
n/a^b^	11153	-	Placenta	-	+	+	+	+
6	16001	+	Lymph node	+	+++	++	++	++
n/a^b^	15009	-	Lymph node	+++	+++	-	+	++
1	15144	+	Lung	-	+	+++	+	+++
n/a^b^	16113	-	Lung	-	++	-	++	++
2	15160	+	Colon	+	+	+++	+	++
n/a^b^	16113	-	Colon	+	+	-	+	+

a Immunostaining: -, negative; +, mild or scattered; ++, moderate; +++, strong.

b Clinically healthy control animal, case number not applicable (n/a).

## Discussion

In a 5-month yersiniosis outbreak, necrotizing typhlocolitis and lymphadenitis were detected in 23 vervets, of which 20 had positive bacterial cultures for *Yersinia enterocolitica*. Of these, nine were found dead, and 14 were euthanized following a rapid clinical decline, typically of no more than 2 days. Yersiniosis in humans often causes mild gastroenteritis and regional lymphadenitis, although more severe disease occurs with some strains, in aged individuals, and with immune compromise (17). In contrast to the classic presentation of gastrointestinal lesions, the index case in this outbreak was a 2-month-old vervet with suppurative tracheitis and extensive pneumonia. Although, this vervet had no evidence of gastrointestinal involvement, there was systemic involvement. Therefore, aerogenous or other modes of transmission could be a possibility. Primary pneumonia due to *Y. enterocolitica* is exceedingly rare in immune-competent humans, with only 18 reported cases, 11 of which had no enteral involvement (18). Furthermore, most cases identified in vervets at WFSM had necrotizing lesions with bacterial colonies outside the gastrointestinal tract (70%), most often in the liver, spleen, and lungs.

The mechanism by which *Y. enterocolitica* infects the gastrointestinal tract is relatively well described. Pathogenic *Y. enterocolitica* strains cross the intestinal mucosal border by attaching to and transporting through intestinal microfold cells (M cells) *via* a β1-integrin reaction with the *Yersinia* invasion virulence factor (InvA) (19). The bacteria replicate extracellularly into microcolonies (20). Through the role of *Yersinia* outer membrane proteins, the bacteria can resist phagocytosis, suppress oxidative bursts, and inhibit proinflammatory cytokine production, including interleukin-8 secretion (21). The extracellular proteins are produced by the type III secretion system pathway, which is encoded by the pYV plasmid (22). Early during the infection period with highly virulent strains, intestinal macrophages, dendritic cells, and neutrophils may facilitate the lymphatic spread of the bacteria to regional lymph nodes or hematogenous spread to the liver, spleen, and lung (23). *In vitro* infection of isolated human dendritic cells with *Y enterocolitica* induced maturation, indicated by upregulation of CD83 and CD86, but downregulated MHC II expression and impaired T cell proliferation up to 6 days post-infection (24). As *Y. enterocolitica* can resist innate immunity, a robust adaptive immune response, including T cells, is needed for protection and, when delayed, promotes increased morbidity and mortality (25, 26).

The immunohistochemical results from affected vervets indicated a robust innate response, including infiltration of neutrophils (MPO) and macrophages (HAM56) compared to age-matched negative control tissues, but little to no adaptive T (CD3) or B (CD20) cell responses. These findings correlate with humans and experimental studies in mice (27, 28). Recent studies point out that *Yersinia* targets innate immune cells, including neutrophils and dendritic cells, using *Yersinia* outer proteins (Yops) to establish tissue infection (27, 29).

Different studies in mouse models suggest that although the bacteria are susceptible to neutrophil-mediated killing initially, the intimate interaction with neutrophils during the infection provides an environment to sustain the infection (27, 30). Studies in humans and mice using different species of *Yersinia* show that recruited neutrophils express abundant inducible nitric oxide synthase (iNOS) (28, 30, 31). There is increasing evidence that suggests iNOS induces adaptive immunosuppression. In septic shock patients, neutrophils with arginase expression impair T cell function (32, 33). Furthermore, myelomonocytic-derived cells of the innate immune system can modulate immunosuppressive responses in the adaptive immune system, thus resulting in T cell depletion and impaired bacterial clearance (34). The robust neutrophilic response may have contributed to the impaired T cell response noted in these vervets.

In addition, experimental mouse studies showed that *Y. enterocolitica* differentially targets splenic dendritic cell subpopulations, which was associated with reduced T cell proliferation (29). The antigen-presenting cell (APCs/CD11c) population increases in the *Y. enterocolitica*-infected vervets in this study. The increase in APCs may suggest *Yersinia* infection in vervets induces tolerant dendritic cells and dampens T cell-mediated response. This additional mechanism may have contributed to the suppression of adaptive response. Further studies could unveil the underlying mechanism of immune suppression. Overall, the lack of an adaptive immune response may explain why *Y. enterocolitica* was able to cause fulminant infection with rapid systemic dissemination in the vervets.

Pregnancy is associated with a shift in placental immune defense that permits embryonic implantation (35). Although the fetus was unaffected, one pregnant vervet in the colony succumbed to systemic yersiniosis, including placentitis. In mice and pigs, infant and maternal mortality caused by *Y. enterocolitica* infection is variable and influenced by pregnancy trimester at exposure (36, 37). The pregnant vervet described in this study became infected with *Yersinia*, which spread to the placenta, resulting in placentitis. It is unknown during which trimester or how long the dam had been infected before death. Additional cases of placental involvement were not identified. The immunologic mechanisms behind pregnancy-related susceptibility to *Y. enterocolitica* remain unknown.

The source of the infection has not been determined at the time of this publication. The possibility of asymptomatic *Yersinia enterocolitica* carriers within the colony is suspected, as cases occurred sporadically when the colony was located in California. Of the 23 cases reported here, five animals originated from the previous facility, and 18 were born at Wake Forest. The dam of the index case originated from the California facility but did not develop yersiniosis or culture positive.

Due to the lack of fresh isolates from the outbreak, attempts to serotype the bacteria from DNA derived from formalin fixed paraffin embedded (FFPE) samples were negative. We considered the possibility that the genomic DNA from FFPE sections was either fragmented/degraded or severely crosslinked by aldehyde fixation, or the amount of bacterial DNA was diluted by mammalian DNA (below the level of detection) or the bacterial strain represents a different type other than what our primer/probe sets were designed to detect.

The reason for the rapid spread of the infection through both WFSM Vervet Research Colony buildings was unclear. Aerosolization of the organisms in the feces during spray cleaning was a possible mechanism. Biosecurity protocols limited access to buildings, and authorized personnel were required to change personal protective equipment between pens. Potential sources of infection include rodents, small mammals, and birds that live around the colony. Cases described in a recent yersiniosis outbreak in Caribbean vervets had a more classical presentation with a prevalence of gastrointestinal lesions. In that outbreak, contact with local wildlife was suspected to be minimal, but small wild mammals, birds, and amphibians were regularly observed in the vicinity (38). Food and water sources are commonly implicated in transmission and spread in humans (39). The same food and water sources were provided to rhesus and cynomolgus macaques housed on the same premises as the vervets, some immune suppressed, yet none developed the disease. Institutions typically report high morbidity and mortality in vervets infected with *Y. enterocolitica*, suggesting they are more susceptible than macaques (38). While *Yersinia enterocolitica* strains have been isolated from macaques, they have not been associated with significant clinical disease (38, 40, 41). Additional factors, including an alkaline environment and ambient temperatures, may have contributed to the virulence of *Yersinia* pathogens (42).

Additional studies in vervets could provide more information regarding *Yersinia*-induced pathogenesis and adaptive immune system suppression. These studies could have a translational impact on chronic and systemic infections in humans.

## Data availability statement

The original contributions presented in the study are included in the article/supplementary material, further inquiries can be directed to the corresponding author.

## Ethics statement

The animal study was reviewed and approved by Wake Forest School of Medicine International Animal Care and Use Committee (IACUC).

## Author contributions

GB, HA, RA, KM, AH, AL, QW, MG, SA, and MJ analyzed the data and drafted the manuscript. RY, NK, and DC conceded the revised study. GB, HA, RA, KM, and AH conducted necropsy and histology exams. All authors contributed to the article and approved the submitted version.

## Funding

The authors gratefully acknowledge the use of the services and facilities of the Comparative Pathology Laboratory and Virtual Imaging Core in the Department of Pathology, funded by NCI Cancer Center Support Grant (P30CA012197) and the North Carolina Biotechnology Center (no. 2015-IDG-1006). Funding was also provided by the ARP and the NIH-sponsored T32 fellowship Grant, OD010957. The Vervet Research Colony was supported by NIH P40OD010965.

## Conflict of interest

The authors declare that the research was conducted in the absence of any commercial or financial relationships that could be construed as a potential conflict of interest.

## Publisher's note

All claims expressed in this article are solely those of the authors and do not necessarily represent those of their affiliated organizations, or those of the publisher, the editors and the reviewers. Any product that may be evaluated in this article, or claim that may be made by its manufacturer, is not guaranteed or endorsed by the publisher.
